# Supplementary material for: Evaluation of Long-Term Adaptive Immune Responses Specific to SARS-CoV-2: Effect of Various Vaccination and Omicron Exposure
Source: Vaccines (Basel). 2024 Mar 13;12(3):301. doi: 10.3390/vaccines12030301 (PMC10974805; doi:10.3390/vaccines12030301)
Supplement: Supplementary file 1 [file vaccines-12-00301-s001.zip › vaccines-2899603-supplementary.pdf]

Supplementary Table S1. Characteristics of the study populations

	Total (n=78)	Infection-naïve (n=9)	Breakthrough infection (n=69)	*P*-value[a]
**Baseline demographics**				
Age, median (IQR)	46.0 (37.0-52.0)	47.0 (43.8-51.5)	45.0 (37.0-52.0)	0.7013[b]
Female, n (%)	57/78 (73.1%)	9/9 (100%)	48/69 (69.6%)	0.0544[c]
**Comorbidities**, n (%)	20 (25.6%)	3/9 (33.3%)	17/69 (24.6%)	0.6867[c]
Hypertension	9	1	8	
Diabetes mellitus	6	1	5	
Cardiac disease	1	0	1	
Malignancy	4	1	3	
Pulmonary disease	4	1	3	
Hypothyroidism.	1	0	1	
**Information related to COVID-19**				
**Primary series**				0.8027[c]
ChAd	64/78 (82.1%)	8/64 (12.5%)	56/64 (87.5%)	
BNT	12/78 (15.4%)	1/12 (8.3%)	11/12 (91.7%)	
mRNA-1273	2/78 (2.6%)	0/2 (0%)	2/2 (100%)	
**Total vaccine doses**				
2 doses	4/78 (5.1%)	0/9 (0%)	4/69 (5.8%)	1.0000[c]
3 doses	57/78 (73.1%)	5/9 (55.6%)	52/69 (75.4%)	0.2406[c]
4 doses	16/78 (20.5%)	3/9 (33.3%)	13/69 (18.8%)	0.3797[c]
5 doses	1/78 (1.3%)	1/9 (11.1%)	0/69 (0%)	0.1154[c]
**Interval from second dose administration, median in days (IQR)**				
T1	26.0 (23.0-29.0) (n=77)	26.0 (23.0-28.0) (n=9)	26.0 (22.0-29.0) (n=68)	0.8548[b]
T2	161.5 (160.0-165.0) (n=58)	165.0 (162.5-167.3) (n=5)	161.0 (160.0-165.0) (n=53)	0.1430[b]
T3	336.0 (332.3-338.0) (n=59)	334.0 (333.0-336.0) (n=6)	336.0 (331.8-338.3) (n=53)	0.4264[b]
T4	744.0 (736.0-749.0) (n=78)	742.0 (731.5-744.0) (n=9)	744.0 (739.0-750.0) (n=69)	0.0794[b]

Abbreviations: IQR, interquatile range; COVID-19, coronavirus disease 2019; ChAd, ChAdOx1 nCoV-19; BNT, Pfizer-BioNTech; mRNA-1273, Moderna mRNA-1273

[a]*P*-value was calculated by comparing naive and breakthrough infected individuals.

[b]*P*-value by Mann-Whitney test

[c]*P*-value by chi-square or Fisher's exact test

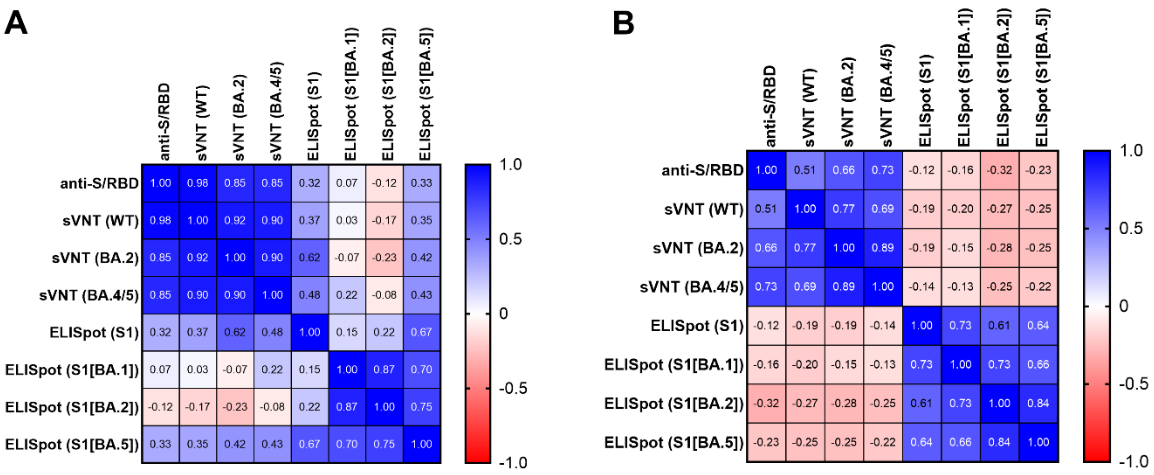

Supplementary Figure S1. Correlogram of SARS-CoV-2-specific adaptive immune measures of the samples collected at T4.

Correlation of SARS-CoV-2 specific immunogenicity parameters measured at T4 in infection-naïve participants (A) and participants experienced breakthrough infection (B).
